# Accuracy of Apple Watch to Measure Cardiovascular Indices in Patients with Cardiac Diseases: Observational Study

**DOI:** 10.5334/gh.1456

**Published:** 2025-09-02

**Authors:** Alaa Abdulhafiz Khushhal, Ashraf Abdelaal Mohamed, Mahmoud Elshahat Elsayed

**Affiliations:** 1 Department of Medical Rehabilitation Sciences, Faculty of Applied Medical Sciences, Umm Al-Qura University, Makkah, Saudi Arabia; 2 Cardiology department, Faculty of Medicine, Al Azhar University, Egypt

**Keywords:** Apple Watch, validity, cardiac diseases

## Abstract

The validity of Apple Watch for measuring heart rate (HR) and oxygen saturation (SpO_2_) in patients with cardiac diseases is still unclear. Therefore, this study aimed to investigate the accuracy of the Apple Watch in measuring HR and SpO_2_ in patients with cardiac diseases. A cross-sectional study recruited 260 cardiac patients, including 190 with regular heart rhythm and 70 with cardiac arrhythmia. Each patient wore the Apple Watch alongside a Polar HR monitor at rest, during and after mild- to moderate-intensity exercise sessions, and wore the Apple Watch alongside a Contec pulse oximeter at rest and after exercise. The Apple Watch showed excellent validity (ICC = 0.100) in measuring the HR at rest, during mild- to moderate-intensity exercise, and after exercise in cardiac patients, as well as in measuring SpO_2_ at rest (ICC = 0.100) and after exercise (ICC = 0.92). However, the validity of the Apple Watch for measuring SpO_2_ decreased slightly after exercise (ICC = 0.85; good validity), especially in patients with an irregular heart rhythm. Overall, the Apple Watch appears valid for measuring HR and SpO_2_ at rest and after exercise, and for measuring HR during mild- to moderate-intensity training in cardiac patients.

## Introduction

Measuring the heart rate (HR) is important to estimate the suitable exercise training intensity and to determine the recommended exercise levels (1). Exercise is one of the important elements to treat cardiac disease, which is a leading cause of death worldwide (2,3,4). The 12-lead electrocardiogram (ECG) is the gold standard to measure HR, but it may not be suitable for use outside the laboratory; therefore, using the Polar chest strap is a practical alternative to be used outside the lab, and it has been tested against the 12-lead ECG during exercise and at rest in both healthy subjects (5,6,7) and cardiac patients (8). The HR following exercise is called HR recovery and it is important to predict mortality risk and cardiorespiratory fitness (9).

There is a linear relationship between HR and oxygen consumption (10). Monitoring oxygen saturation (SpO_2_) is recommended for patients with hypoxemia to help extend their survival (11). Some cardiac patients have hypoxemia, particularly after cardiac arrest (12,13,14). A pulse oximeter is the gold standard for measuring SpO_2_ (15). The Contec CMS50DL pulse oximeter meets the International Organization for Standardization (ISO) criteria for validity (16) and is approved by the Food and Drug Administration (FDA) (17).

All versions of the Apple Watch can measure HR, but the latest versions (Series 6 and 7) can also measure SpO_2_. Photoplethysmography (PPG) is a non-invasive measurement technique used to evaluate HR via a wrist-worn device by detecting changes in blood flow and its associated color changes with each heartbeat. The PPG has been used to measure SpO_2_ and blood pressure in various medical devices (18). PPG technology may also help in the early detection of some cardiovascular diseases such as atherosclerosis and atrial fibrillation (AF) (19).

The new feature of measuring the blood SpO_2_ is available only in the Apple Watch Series 6 and 7. To date, only one study has examined the validity of the Apple Watch compared to two pulse oximeters for measure SpO_2_ at rest and HR during exercise (*r* = 0.995, *P* < 0.001) in 100 participants, including 16 healthy subjects and 84 patients with medically controlled pulmonary diseases [23 patients with chronic obstructive pulmonary disease (COPD) and 61 patients with interstitial lung diseases]. They found that the Apple Watch showed a strong positive correlation with the pulse oximeter for SpO_2_ (*r* = 0.81, *P* < 0.0001) (20). Moreover, the study suggested testing the validity of the Apple Watch for measuring blood SpO_2_ in other groups of patients. A systematic review of recent studies investigating the accuracy of the Apple Watch in measuring SpO_2_ in children and adults found that the Apple Watch has no strong systematic bias. However, no study has specifically investigated the accuracy of the Apple Watch in measuring SpO_2_ in cardiac patients with sinus rhythm and irregular HR at rest and after exercise, or the impact of skin color on measuring SpO_2_. The review suggests that further research is needed in this area (21). Spaccarotella et al. measured SpO_2_ and HR (*r* = 0.98, *P* < 0.0001) during exercise in cardiac patients with sinus rhythm, but there were no cardiac patients with irregular HR, and they did not measure the accuracy of the Apple Watch in measuring SpO_2_ after exercise, or the impact of skin color while measuring SpO_2_ (22). Another recent study measured HR during mild- to moderate-intensity exercises and SpO_2_ at rest in patients with chronic diseases such as diabetes, hypertension, and dyslipidemia. They found that the Apple Watch was accurate to measure HR during exercise (*r* = 0.99, *P* < 0.001) and SpO_2_ at rest (*r* = 0.92, *P* < 0.001) in patients with chronic diseases and recommends validating these findings with other patient groups, such as cardiac patients (23). The rationale for evaluating the Apple Watch in cardiac patients is that they need to measure HR and SpO_2_ to assess and quantify cardiovascular risk and to monitor cardiovascular responses in day-to-day clinical cardiac practice (22). Therefore, this study aimed to evaluate the accuracy of the Apple Watch for measuring HR at rest, during, and after exercises, and to measure SpO_2_ at rest and after exercises in patients with cardiac diseases with both normal and abnormal HR patterns.

## Methods

A cross-sectional study recruited 260 male patients with cardiac diseases (mean [SD]; age 47 (15) years), including 190 ischemic patients with regular heart rhythm and 70 patients with irregular rhythm, and they all completed the study. Out of 260 patients, 160 patients had white skin, 100 had brown skin, and no patient had black skin. This study was approved by the biomedical research ethics committee at Umm Al-Qura University (HAPO-O2-K-012-2022-01-910). The study followed the principles of the Declaration of Helsinki of 1975, revised in 2000, and was conducted at the Umm Al-Qura University Medical Center. This study was registered at ClinicalTrails.gov (NCT05199844). Each patient signed a written consent approving their volunteering to participate in this study and agreeing to the publication of the results.

### Devices and data collection

After medical counseling and physician referral, each participant wore a Polar chest strap (H10, Polar Electro, OY, Finland) and an Apple Watch (Series 8, watchOS 9.0, Apple Inc., California, USA) on the left wrist, which was connected to an iPhone (iPhone 11, iPhoneOS 16.1, Apple Inc., California, USA) (all patients rested their hands on the plastic handrails of the cycle) at rest for 5 min, during exercise for 16 min, and after exercise for 3 min. The exercise session was for 16 min in duration at mild- to moderate-intensity exercise [40%–70% of heart rate reserve (HRR)] according to the patient’s ability using a cycle ergometer (Longstyle, China). The patient wore a Contec pulse oximeter (CMS50DL, Contec Medical Systems Co., Ltd., China) and an Apple Watch at rest and after exercise to measure SpO_2_.

The HR was recorded every 30 s during the whole period and the oxygen saturation was recorded five times at rest and after exercise simultaneously from the Apple Watch and the Polar chest strap and from the Apple Watch and the Contec pulse oximeter, respectively, to obtain the data.

The exercise intensity during the session was calculated based on the HRR. The HRR was calculated as: (maximum HR – resting HR) + resting HR. The maximum HR was calculated based on the following equations: for cardiac patients with no β-blocker, maximum HR = 206.9 – (0.67 × age) (24), and for cardiac patients with β-blockers, maximum HR = 164 – (0.7 × age) (25).

*Inclusion criteria*: cardiac patients with myocardial infarction (6 weeks post-insult), coronary bypass graft surgery (CABG), valve diseases, stable AF, heart failure (HF)-I, II, and III based on the New York Heart Association classification of heart failure (26), and a resting ejection fraction (EF) >50%.

*Exclusion criteria*: cardiac patients with unstable angina, uncontrolled high blood pressure, unstable arrhythmia, the presence of complex ventricular arrhythmias, ST-segment depression ≥2 mm from baseline during exercise testing or recovery, and pacemaker patients.

### Sample size

The suitable number of participants was calculated through the online G-Power program (https://download.cnet.com/GPower/3000-2054_4-10647044.html), considering the alpha error probability = 0.05, power = 0.95, and Cohen’s *f* ‘effect size’ = 0.23 (small effect size) (27), resulting in a total sample size of 248 participants to provide reliable results. An additional 12 participants were added to compensate for any drop or withdrawal. Additionally, the sample size required to clarify significant differences in the present study was chosen considering the guidelines of the past studies in the same field (28,29,30,31,32,33,34,35).

### Data analysis

All data were analyzed using SPSS version 29 (IBM Corp, Chicago, IL, USA). The data are presented as the mean and standard deviation. The Shapiro–Wilk test was used to verify the normal distribution of the data. The mean difference (MD) and standard deviation of the mean difference (SDD) were calculated to construct Bland–Altman plots. Bland–Altman plots were used to test the bias (MD) and the limits of agreement (LoA, MD ± 1.96 * SDD) of the data. Intraclass correlation coefficients (ICCs) were used to determine the correlation between the Apple Watch and Polar chest strap for the HR variable and between the Apple Watch and Contec pulse oximeter for the SpO_2_ variable. The strength of the ICC was interpreted based on the Fokkema et al. suggestion as follows: ICC >0.90 was excellent, 0.75 to 0.90 was good, 0.60 to 0.75 was moderate, and <0.60 was low (28). An independent *t*-test was performed to calculate the difference between white and brown skin color patients for the SpO_2_ variable at rest. The *P* value was set at 0.05 for significant results.

## Results

### Heart rate

**Figure 1:**
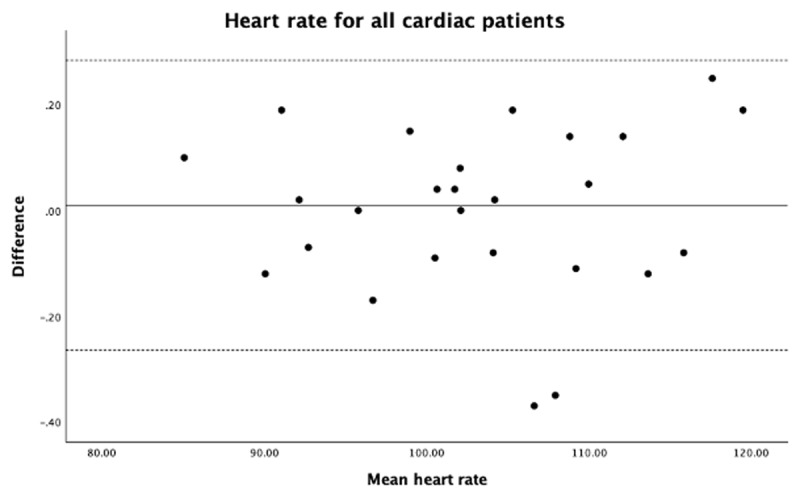
Heart rate for all cardiac patients.

There was an excellent correlation for HR measurements in patients with regular and irregular rhythms and for overall cardiac patients (Table 1). The LoA were (upper, lower LoA) 0.28, –0.26, and the bias (mean difference) was 0.01 for HR all in cardiac patients with no outlier (Figure 1).

**Table 1:** Intraclass correlation for Apple Watch in cardiac patients with regular and irregular rhythm in HR and oxygen saturation at rest and after exercise.


OUTCOMES	ICC (95% CI)	*P* VALUE	MEAN (SD) OF PRACTICAL (APPLE WATCH)	MEAN (SD) OF CRITERION (POLAR)

**Heart rate**				

Cardiac patients with regular rhythm	0.100 (0.100–0.100)	<0.001	102.00 (8.06)	101.97 (8.03)

Cardiac patients with irregular rhythm	0.100 (0.100–0.100)	<0.001	106.67 (9.70)	106.63 (9.73)

All cardiac patients	0.100 (0.100–0.100)	<0.001	103.25 (8.77)	103.24 (8.75)

			**MEAN (SD) OF PRACTICAL (APPLE WATCH)**	**MEAN (SD) OF CRITERION (CONTEC)**

**SpO_2_ at rest**				

Cardiac patients with regular rhythm	0.98 (0.94–0.99)	<0.001	97.04 (1.09)	96.91 (0.99)

Cardiac patients with irregular rhythm	0.99 (0.93–0.100)	<0.001	96.86 (0.84)	96.80 (0.73)

All cardiac patients	0.98 (0.95–0.99)	<0.001	96.99 (1.08)	96.88 (0.91)

**SpO_2_ after exercise**				

Cardiac patients with regular rhythm	0.94 (0.79–0.97)	0.02	97.54 (0.84)	97.33 (0.75)

Cardiac patients with irregular rhythm	0.85 (0.15–0.97)	<0.001	97.14 (0.78)	97.03 (0.50)

All cardiac patients	0.92 (0.80–0.97)	<0.001	97.27 (0.69)	97.43 (0.83)


CI, confidence interval; ICC, intraclass correlation coefficient.

### Oxygen saturation (SpO_2_) at rest and after exercise

**Figure 2:**
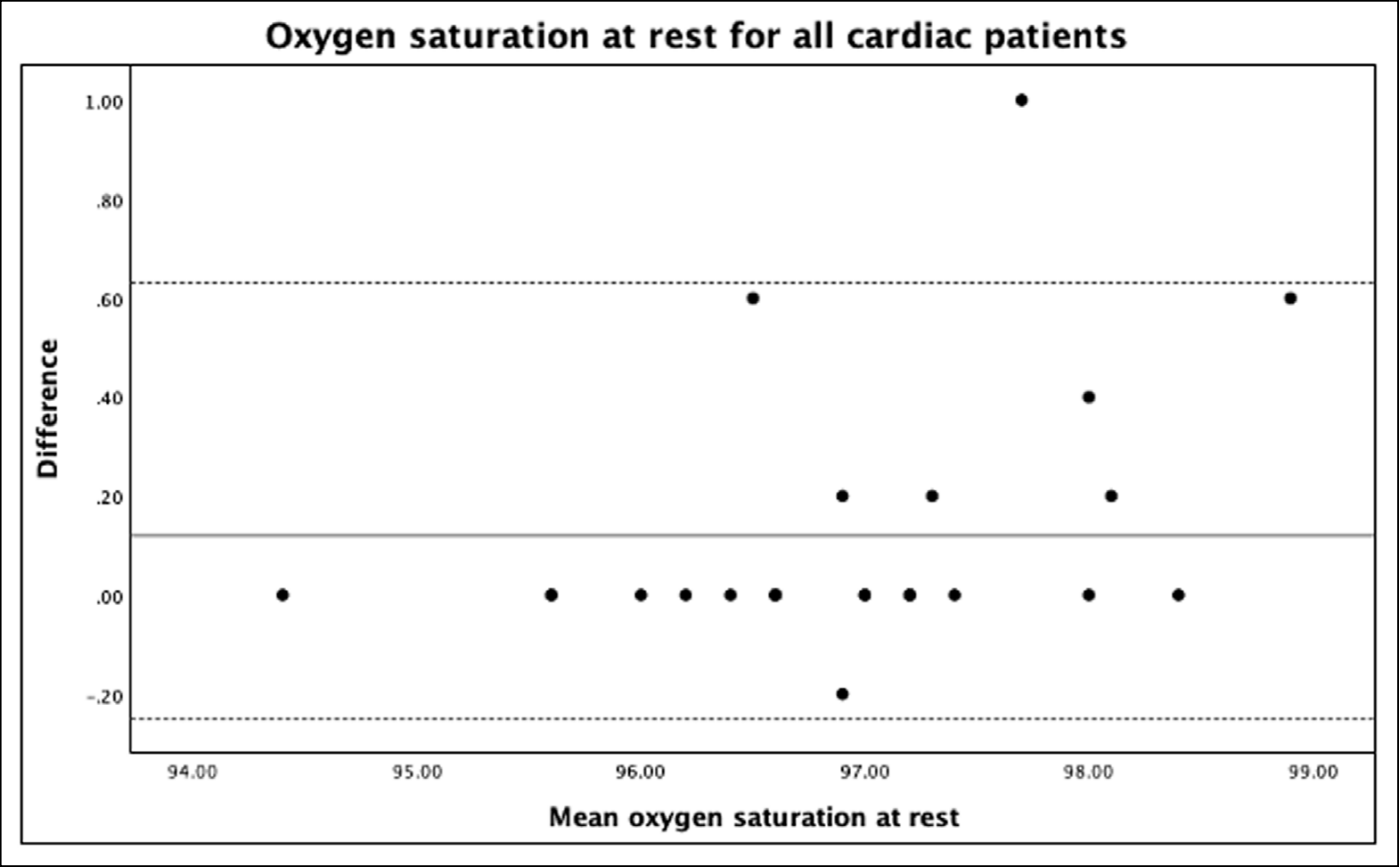
Oxygen saturation of all cardiac patients at rest.

**Figure 3:**
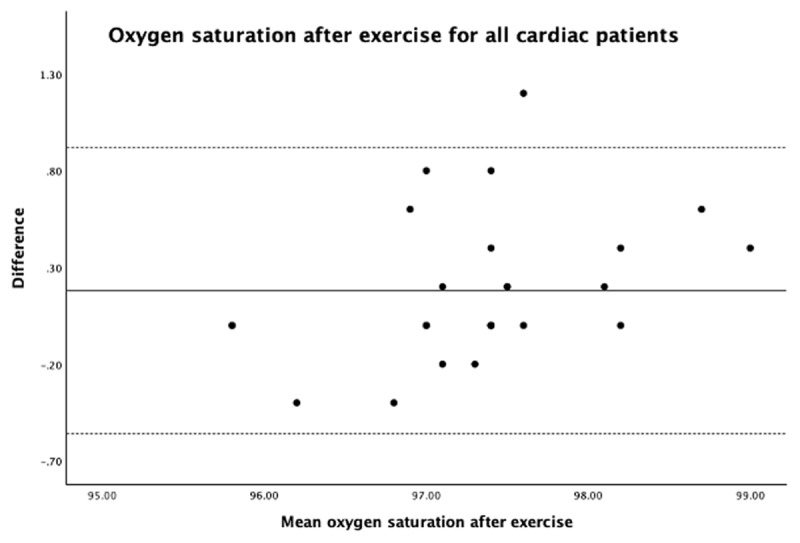
Oxygen saturation of all cardiac patients after exercise.

Oxygen saturation had an excellent correlation with all cardiac patients, including those with regular and irregular heart rhythms at rest (Table 1). The LoA was 0.63, –0.25. The mean difference was 0.12 for all cardiac patients at rest with an outlier of <4% SpO_2_ (Figure 2). Oxygen saturation after exercise had an excellent correlation with all cardiac patients and in patients with regular heart rhythm, but there is a slight reduction in patients with irregular heart rhythms, indicating good correlation (Table 1). Overall, the correlation for SpO_2_ was less after exercise than at rest in all cardiac patients (Table 1). The LoA after exercise was 0.86, –0.50, and bias (mean difference) was 0.16 with an outlier of <4% SpO_2_ (Figure 3). There was no statistically significant difference between white and brown skin color patients in SpO_2_ at rest (*P* = 0.498).

## Discussion

Mobile technology has grown tremendously in the last decade, with modern healthcare is shifting its focus to incorporate mobile health technology. The ability to measure HR using wrist-worn devices facilitates the continuous monitoring of patient status and cardiovascular responses (28). Wearable technologies can be used for detecting and diagnosing health problems in patients with cardiac diseases and may help to manage and improve patient care outcomes (36). Such technology is acceptable to measure HR in daily clinical practice and to detect cardiac diseases such as irregular heart rhythms (37).

Interest in validating the accuracy of mobile health technology in the modern health care of patients with cardiac diseases has tremendously increased in the past few years. In this study, the HR and oxygen saturation were evaluated using the Apple Watch compared to gold-standard devices in 260 male cardiac patients at rest, during, and after mild- to moderate-intensity exercise sessions. For HR, the results showed excellent correlations in cardiac patients with both regular and irregular rhythms throughout the period of the exercise session, including rest, during, and after exercise. For oxygen saturation, the Apple Watch showed excellent correlation at rest for all cardiac patients with regular and irregular rhythms, with <4% outliers, and after exercise sessions in cardiac patients with regular rhythm, whereas a slightly lower (good) correlation was observed after exercise sessions in cardiac patients with irregular rhythm.

Precise monitoring of the HR is important to prescribe exercise intensity for patients with cardiac disease (38). The results of the current study indicate that the Apple Watch can be a convenient and satisfactory tool to evaluate HR in patients with cardiac disease at rest and during mild- to moderate-intensity exercise. These findings are align with earlier studies that reported promising results regarding the accuracy of the Apple Watch in monitoring cardiovascular metrics in cardiac patients during rest and training. This study provides future directions to confirm these findings and to recommend the Apple Watch as an accurate monitoring device in cardiac rehabilitation settings (39).

Our results also support the use of the Apple Watch in daily clinical practice (37). Although the accuracy of using the wrist-worn Apple Watch in monitoring the HR was previously evaluated in healthy subjects (40,28,41,42,43,44,30,29), there is still an ongoing urgent need to emphasize its validity and accuracy in clinical practice (39,28,45).

Because of the increasing tendency of cardiac patients to rely on wrist-worn devices to monitor their HR and guide safe training intensity during cardiac rehabilitation, appropriate validation and accurate evaluation of wrist-worn devices use in the cardiac rehabilitation field is imperative (41) to stay within the recommended safe activity level and HR range during training sessions.

Regarding HR, the results of the current study were in accordance with those of Nelson et al., who reported that the accuracy of using the Apple Watch in monitoring the 24-h HR period is useful for monitoring cardiac activities in clinical settings (46). Prior research findings reported acceptable error rates when monitoring HR using the Apple Watch under controlled conditions (24). A study of 50 cardiac patients (half with AF and the other half with normal rhythm) measured HR three times a day using the Apple Watch and telemetry during rest for 2 days. The results found that the HR accuracy in AF patients (*r* = 0.86) was higher than in cardiac patients who had normal rhythm (*r* = 0.64); the overall accuracy was 0.70. However, more studies with large sample sizes are needed to confirm these findings (47). Moreover, the accuracy of the Apple Watch in measuring HR during exercise is still unknown for cardiac patients with normal rhythm and AF.

A recent study measured the HR in 80 cardiac patients, including some patients with AF, in phase II or III of cardiac rehabilitation (exercises including steady cycling and treadmill). This study compared HR from the limb leads of the ECG with the Polar HR, Apple Watch, Fitbit Blaze, Garmin Forerunner 235, and TomTom Spark Cardio (one device on each wrist), which means that only 40 patients were tested with the Apple Watch. They recorded HR at rest and at 3, 5, and 7 min of cycling and treadmill training. They found that the Polar chest strap (*r* = 0.99) was the most accurate during all exercises compared to the ECG, followed by the Apple Watch (*r* = 0.80), and the accuracy of the Apple Watch during cycling increased to 0.89 (8). However, the proportion of AF in 80 patients was low (only 12 AF) as they were mixed with the normal HR patients while testing, so it is not possible to know if the AF affected the accuracy of the Apple Watch and further research is needed in this area. The eight-time points measured in this study were good, but if the study measured the HR every 15 or 30 s, the results could be more meaningful. Falter et al. reported a clinically acceptable overall accuracy of the Apple Watch in monitoring HR in patients with cardiovascular disease: moderate correlation [ICC = 0.729 (*P* < 0.001)] between the Apple Watch and the gold standard electrocardiogram at rest, good correlation [ICC = 0.828 (*P* < 0.001)] during moderate-intensity training, and excellent correlation [ICC = 0.958 (*P* < 0.001] during high-intensity training (39).

Using an Apple Watch in the hospital is cheaper and easier than using an ECG in daily clinical practice. Wang et al. found excellent correlation coefficients (*rc* = 0.91) when comparing the efficacy of a number of wrist-worn devices, including the Apple Watch 3 against the Polar H7 chest strap monitor in healthy subjects (41). Etiwy et al. found good correlation coefficients (*rc* = 0.81) between wrist-worn devices, including the Apple Watch 3 and both the ECG and the Polar H7 chest strap monitor during cardiac rehabilitation (phases II and III) in patients with cardiovascular disorders (8). Seshadri et al. found good agreement (*rc* = 0.86) between the Apple Watch and telemetry electrocardiogram in evaluating the AF patients’ HR (44) that supports the current study findings.

Previous research reported greater accuracy by the Apple Watch in monitoring the HR compared to other wearable devices (28,30,41), with limited overall error (32,28,48), higher agreement with the ECG gold standard procedure (31,38), and an acceptable error range even over an extended 24-h monitoring period (48).

Regarding SpO_2_, although the Apple Watch showed consistency and concordance with medical-grade pulse oximeters during the SpO_2_ evaluation in adults, more research is needed to resolve many accuracy-related concerns (21). The outliers in the previous studies were up to 15% SpO_2_, but the current study found a lower percentage of outliers, which is <4% SpO_2_, and the possible reason for this could be the controlled rest situation with the supported wrist in an armchair was recommended by the manufacturer while measuring SpO_2_ in an Apple Watch (48).

The results of the current study showed that the Apple Watch can be considered for monitoring SpO_2_ in patients with cardiac diseases both at rest and post-exercise. These results are in line with earlier results by Spaccarotella et al., who reported that the standard measuring device and the Apple Watch in monitoring SpO_2_ in healthy subjects and in patients with cardiovascular disease at rest are similar (22). Also, the earlier research reports provide details about the accepted accuracy of the Apple Watch in monitoring the SpO_2_ changes under normal and hypoxic conditions in healthy adults.

The difference in the current study results between the accuracy of the Apple Watch at rest and after exercise, particularly in cardiac patients with an irregular HR, could be due to the unstable HR influenced the results of SpO_2_. According to the manufacturer, the patients should to be in a complete rest with HR <150 beats per minute to get the accurate measurement of SpO_2_ (48).

On the other hand, a study conducted by Pätz et al. failed to provide a clear results about the accuracy of the Apple Watch measuring SpO_2_ due to improper technique and use of watch by the adult and child participants resulted in multiple incorrect readings (49).

The Fitzpatrick skin type scale’s impact on measuring SpO_2_ using the Apple Watch is still unclear (23). Fortunately, no obvious variability exists between participants in the skin color of patients with COVID, and these findings are in line with the current study results (*P* = 0.498), as this is the first study to examine the difference between white and brown skin color cardiac patients using an Apple Watch with regular and irregular heart rhythms, which limits the negative contributing factors since SpO_2_ accuracy can be influenced by skin pigmentation (50). Further studies in different populations are needed to confirm this finding.

## Limitations

The maximum HR was estimated in this study, but this is due to the difficulty of doing a real test, such as a stress test to obtain the real maximum HR in patients with cardiac diseases. Inclusion of the male gender only limits the broad generalizability of the current study results in clinical practice. However, there is no study showing any significant difference between male and female yet. Additionally, this study measured SpO_2_ in patients with white and brown color skin tones with no representation from those with dark color skin. Further studies are needed to address these limitations and use more standardized evaluation procedures.

## Conclusions

This study provides the first evidence for the accuracy of the Apple Watch in monitoring HR and SpO_2_ in cardiac patients. These findings suggest the Apple Watch may be suitable in day-to-day clinical cardiology practice for monitoring cardiovascular responses and assessing symptoms and risks in cardiac patients.

The Apple Watch demonstrated acceptable accuracy in monitoring HR in cardiac patients with both regular and irregular rhythms. Supported by earlier studies, our study results recommend the use of Apple Watch for monitoring cardiovascular responses in cardiac patients.

## Data Accessibility Statement

The data that support the findings of this study are available from the corresponding author upon reasonable request.

## References

[1] Warren JM, Ekelund U, Besson H, Mezzani A, Geladas N, Vanhees L, et al. Assessment of physical activity – a review of methodologies with reference to epidemiological research: A report of the exercise physiology section of the European Association of Cardiovascular Prevention and Rehabilitation. European Journal of Cardiovascular Prevention and Rehabilitation. 2010;17(2):127–139. DOI: 10.1097/HJR.0b013e32832ed875 20215971

[2] Odah MM, Alfakieh HO, Almathami AA, Almuashi IM, Awad M, Ewis AA. Public awareness of coronary artery disease and its risk factors among Al-Qunfudah governorate population. Journal of Umm Al-Qura University of Medical Sciences. 2022;8:34–38. DOI: 10.54940/ms42784839

[3] Abdulrahman OA, Baaqeel RG, Alotaibi NF, Althebeti RR, Bahakeem RF, Tukruni OA, et al. Knowledge and perception of cardiac surgery among medical students in the Western region of Saudi Arabia. The Cureus Journal of Medical Sciences. 2023;15(5):e38605. DOI: 10.7759/cureus.38605 PMC10239667 37284372

[4] Shammah AA. Umm Al Qura University students ideas about adult cardiopulmonary resuscitations. Journal of Clinical and Experimental Cardiology. 2021;11:694.

[5] Lee CM, Gorelick M. Validity of the smarthealth watch to measure heart rate during rest and exercise. Measurement in Physical Education and Exercise Science. 2011;15(1):18–25. DOI: 10.1080/1091367X.2011.539089

[6] Vanderlei LCM, Silva RA, Pastre CM, Azevedo FM, Godoy MF. Comparison of the Polar S810i monitor and the ECG for the analysis of heart rate variability in the time and frequency domains. Brazilian Journal of Medical and Biological Research. 2008;41:854–859. DOI: 10.1590/S0100-879X2008005000039 18853042

[7] Weippert M, Kumar M, Kreuzfeld S, Arndt D, Rieger A, Stoll R. Comparison of three mobile devices for measuring R-R intervals and heart rate variability: Polar S810i, Suunto t6 and an ambulatory ECG system. European Journal of Applied Physiology. 2010;109:779–786. DOI: 10.1007/s00421-010-1415-9 20225081

[8] Etiwy M, Akhrass Z, Gillinov L, Alashi A, Wang R, Blackburn, G, et al. Accuracy of wearable heart rate monitors in cardiac rehabilitation. Cardiovascular Diagnosis and Therapy. 2019;9(3):262. DOI: 10.21037/cdt.2019.04.08 31275816 PMC6603497

[9] Cole CR, Blackstone EH, Pashkow FJ, Snader CE, Lauer MS. Heart-rate recovery immediately after exercise as a predictor of mortality. The New England Journal of Medicine. 1999;341:1351–1357. DOI: 10.1056/NEJM199910283411804 10536127

[10] Crisafulli A, Pittau G, Lorrai L, Carcassi AM, Cominu M, Tocco F, et al. Poor reliability of heart rate monitoring to assess oxygen uptake during field training. International Journal of Sports Medicine. 2005:55–59. DOI: 10.1055/s-2005-837504 16388443

[11] Porte P. Clinical indications for noninvasive positive pressure ventilation in chronic respiratory failure due to restrictive lung disease, COPD, and nocturnal hypoventilation—A consensus conference report. Chest. 1999;116(2):521. DOI: 10.1378/chest.116.2.521 10453883

[12] Kilgannon JH, Jones AE, Shapiro NI, Angelos MG, Milcarek B, Hunter K, et al. Association between arterial hyperoxia following resuscitation from cardiac arrest and in-hospital mortality. JAMA. 2010;303(21):2165–2171. DOI: 10.1001/jama.2010.707 20516417

[13] Al Attar WS, Khushhal A. Soccer-related injuries pre-and post-COVID-19 lockdown in Saudi Arabia: An epidemiological study. The Journal of Sports Medicine and Physical Fitness. 2022;63(5):660–666. DOI: 10.23736/S0022-4707.22.14073-9 36205085

[14] Khushhal A, Alsubaiei M. Barriers to establishing outpatient cardiac rehabilitation in the Western Region of Saudi Arabia: A cross-sectional study. Journal of Multidisciplinary Healthcare. 2023:653–661. DOI: 10.2147/JMDH.S398687 PMC10010741 36923361

[15] Schnapp LM, Cohen NH. Pulse oximetry: Uses and abuses. Chest. 1990;98(5):1244–1250. DOI: 10.1378/chest.98.5.1244 2225973

[16] Lipnick MS, Feiner JR, Au P, Bernstein M, Bickler PE. The accuracy of 6 inexpensive pulse oximeters not cleared by the Food and Drug Administration: The possible global public health implications. Anesthesia & Analgesia. 2016;123(2):338–345. DOI: 10.1213/ANE.0000000000001300 27089002

[17] U.S. Food and Drug Administration. Establishment registration & device listing [Internet]. US: US Department of Health and Human Services. c2024 [updated 2024 Nov; cited 2023 Sep 28]. Available from: https://www.accessdata.fda.gov/scrIpts/cdrh/cfdocs/cfRL/rl.cfm?lid=205276&lpcd=DQA

[18] Allen J. Photoplethysmography and its application in clinical physiological measurement. Physiological Measurement. 2007;28(3):R1. DOI: 10.1088/0967-3334/28/3/R01 17322588

[19] Temko A. Accurate heart rate monitoring during physical exercises using PPG. IEEE Transactions on Biomedical Engineering. 2017;64(9):2016–2024. DOI: 10.1109/TBME.2017.2676243 28278454

[20] Pipek LZ, Nascimento RFV, Acencio MMP, Teixeira LR. Comparison of SpO2 and heart rate values on Apple Watch and conventional commercial oximeters devices in patients with lung disease. Science Reports. 2021;11(1):18901. DOI: 10.1038/s41598-021-98453-3 PMC8460792 34556765

[21] Windisch P, Schröder C, Förster R, Cihoric N, Zwahlen DR, Windisch PY. Accuracy of the Apple watch oxygen saturation measurement in adults: A systematic review. The Cureus Journal of Medical Sciences. 2023;15(2):e35355. DOI: 10.7759/cureus.35355 PMC10039641 36974257

[22] Spaccarotella C, Polimeni A, Mancuso C, Pelaia G, Esposito G, Indolfi C. Assessment of non-invasive measurements of oxygen saturation and heart rate with an Apple Smartwatch: Comparison with a standard pulse oximeter. Journal of Clinical Medicine. 2022;11(6):1467. DOI: 10.3390/jcm11061467 35329793 PMC8951323

[23] Khushhal AA, Mohamed AA, Elsayed ME. Accuracy of Apple watch to measure cardiovascular indices in patients with chronic diseases: A cross sectional study. Journal Multidisciplinary Healthcare. 2024;17:1053–1063. DOI: 10.2147/JMDH.S449071 PMC10941792 38496326

[24] Chewning JM. Understanding aquatic heart rate deductions. Akwamagazine. 2010;32(4):29.

[25] Brawner CA, Ehrman JK, Schairer JR, Cao JJ, Keteyian SJ. Predicting maximum heart rate among patients with coronary heart disease receiving beta-adrenergic blockade therapy. American Heart Journal. 2004;148(5):910–914. DOI: 10.1016/j.ahj.2004.04.035 15523326

[26] Heart Education Assessment Rehabilitation Toolkit. Pathophysiology of acute coronary syndrome and heart failure – classification of heart rate [Internet]. Australia: Heart Online; 2024. Available from: https://www.heartonline.org.au/articles/pathophysiology/pathophysiology-of-acute-coronary-syndrome-and-heart-failure

[27] Cohen J. Statistical power analysis for the behavioral sciences. 2nd ed. New York. Routledge; 1988.

[28] Shcherbina A, Mattsson CM, Waggott D, Salisbury H, Christle JW, Hastie T, et al. Accuracy in wrist-worn, sensor-based measurements of heart rate and energy expenditure in a diverse cohort. Journal of Personalized Medicine. 2017;7(2):3. DOI: 10.3390/jpm7020003 28538708 PMC5491979

[29] Kooiman TJ, Dontje ML, Sprenger SR, Krijnen WP, Van der Schans CP, de Groot M. Reliability and validity of ten consumer activity trackers. BMC Sports Science, Medicine and Rehabilitation. 2015;7:24. DOI: 10.1186/s13102-015-0018-5 PMC4603296 26464801

[30] Wallen MP, Gomersall SR, Keating SE, Wisløff U, Coombes JS. Accuracy of heart rate watches: implications for weight management. PLoS One. 2016;11(5):e0154420. DOI: 10.1371/journal.pone.0154420 27232714 PMC4883747

[31] Bai Y, Hibbing P, Mantis C, Welk GJ. Comparative evaluation of heart rate-based monitors: Apple Watch vs Fitbit Charge HR. Journal of Sports Sciences. 2018;36(15):1734–1741. DOI: 10.1080/02640414.2017.1412235 29210326

[32] Boudreaux BD, Hebert EP, Hollander DB, Williams BM, Cormier CL, Naquin MR, et al. Validity of wearable activity monitors during cycling and resistance exercise. Medicine & Science in Sports & Exercise. 2018;50(3):624–633. DOI: 10.1249/MSS.0000000000001471 29189666

[33] Chowdhury E, Western M, Nightingale T, Peacock O, Thompson D. Assessment of laboratory and daily energy expenditure estimates from consumer multi-sensor physical activity monitors. PLoS One. 2017;12(2):e0171720. DOI: 10.1371/journal.pone.0171720 28234979 PMC5325221

[34] Khushhal A, Nichols S, Evans W, Gleadall-Siddall DO, Page R, O’Doherty AF, Carroll S, et al. Validity and reliability of the Apple Watch for measuring heart rate during exercise. Sports Medicine International Open. 2017;1(06):E206–E211. DOI: 10.1055/s-0043-120195 30539109 PMC6226089

[35] Theurl F, Schreinlechner M, Sappler N, Toifl M, Dolejsi T, Hofer F, et al. Smartwatch-derived heart rate variability: A head-to-head comparison with the gold standard in cardiovascular disease. European Heart Journal of Digital Health. 2023;4(3):155–164. DOI: 10.1093/ehjdh/ztad022 37265873 PMC10232241

[36] Leclercq C, Witt H, Hindricks G, Katra RP, Albert D, Belliger A, et al. Wearables, telemedicine, and artificial intelligence in arrhythmias and heart failure: Proceedings of the European Society of Cardiology Cardiovascular Round Table. Europace. 2022;24(9):1372–1383. DOI: 10.1093/europace/euac052 35640917

[37] Manninger M, Zweiker D, Svennberg E, Chatzikyriakou S, Pavlovic N, Zaman JA, et al. Current perspectives on wearable rhythm recordings for clinical decision-making: The wEHRAbles 2 survey. Europace. 2021;23(7):1106–1113. DOI: 10.1093/europace/euab064 33842972

[38] Mann T, Lamberts RP, Lambert MI. Methods of prescribing relative exercise intensity: Physiological and practical considerations. Sports Medicine. 2013;43:613–625. DOI: 10.1007/s40279-013-0045-x 23620244

[39] Falter M, Budts W, Goetschalckx K, Cornelissen V, Buys R. Accuracy of Apple Watch measurements for heart rate and energy expenditure in patients with cardiovascular disease: Cross-sectional study. JMIR Mhealth Uhealth. 2019;7(3):e11889. DOI: 10.2196/11889 30888332 PMC6444219

[40] Wang R, Blackburn G, Desai M, Phelan D, Gillinov L, Houghtaling P, et al. Accuracy of wrist-worn heart rate monitors. JAMA Cardiology. 2017;2(1):104–106. DOI: 10.1001/jamacardio.2016.3340 27732703

[41] Parak J, Korhonen I. Evaluation of wearable consumer heart rate monitors based on photopletysmography. Conference Proceedings of IEEE Engineering in Medical and Biology Society. 2014;3670–3673. DOI: 10.1109/EMBC.2014.6944419 25570787

[42] Jo E, Lewis K, Directo D, Kim MJ and Dolezal BA. Validation of biofeedback wearables for photoplethysmographic heart rate tracking. Journal of Sports Science and Medicine. 2016;15(3):540. 27803634 PMC4974868

[43] Gillinov S, Etiwy M, Wang R, Blackburn G, Phelan D, Gillinov AM, et al. Variable accuracy of wearable heart rate monitors during aerobic exercise. Medicine & Science in Sports & Exercise. 2017;49(8):1697–1703. DOI: 10.1249/MSS.0000000000001284 28709155

[44] Dooley EE, Golaszewski NM, Bartholomew JB. Estimating accuracy at exercise intensities: A comparative study of self-monitoring heart rate and physical activity wearable devices. JMIR mHealth uHealth. 2017;5(3):e7034. DOI: 10.2196/mhealth.7043 PMC5374271 28302596

[45] El-Amrawy F, Nounou MI. Are currently available wearable devices for activity tracking and heart rate monitoring accurate, precise, and medically beneficial? Healthcare Informatics Research. 2015;21(4):315–320. DOI: 10.4258/hir.2015.21.4.315 26618039 PMC4659890

[46] Nelson BW, Allen NB. Accuracy of consumer wearable heart rate measurement during an ecologically valid 24-hour period: Intraindividual validation study. JMIR mHealth uHealth. 2019;7(3):e10828. DOI: 10.2196/10828 30855232 PMC6431828

[47] Seshadri DR, Bittel B, Browsky D, Houghtaling P, Drummond CK, Desai M, et al. Accuracy of the Apple watch 4 to measure heart rate in patients with atrial fibrillation. IEEE Journal of Translational Engineering Health and Medicine. 2019;8:1–4. DOI: 10.1109/JTEHM.2019.2950397 PMC7049290 32128290

[48] Apple Support. How to use the Blood Oxygen app on Apple Watch [Internet]. Saudi Arabia: Apple Inc; 2024. Available from: https://support.apple.com/en-sa/HT211027

[49] Pätz C, Michaelis A, Markel F, Löffelbein F, Dähnert I, Gebauer RA, et al. Accuracy of the Apple Watch oxygen saturation measurement in adults and children with congenital heart disease. Pediatric Cardiology. 2023;44(2):333–343. DOI: 10.1007/s00246-022-02987-w 35995951

[50] Luks AM, Swenson ER. Pulse oximetry for monitoring patients with COVID-19 at home. Potential pitfalls and practical guidance. Annals of American Thoracic Society. 2020;17(9):1040–1046. DOI: 10.1513/AnnalsATS.202005-418FR PMC7462317 32521167

